# Artificial intelligence (AI) anxiety and nurses’ turnover intention during healthcare digital transformation: the mediating role of occupational fatigue

**DOI:** 10.3389/fpubh.2026.1982743

**Published:** 2026-10-22

**Authors:** Lidan Zhu, Feng Zang, Guangxu Mao, Xiang Li, Xiaofang Shen

**Affiliations:** 1 Party Committee Office, Xinghua People's Hospital Affiliated to Yangzhou University, Xinghua, Jiangsu, China; 2 Department of Infection Control, The First Affiliated Hospital with Nanjing Medical University, Nanjing, Jiangsu, China; 3 Department of Epidemiology and Infection Control, Xinghua People's Hospital Affiliated to Yangzhou University, Xinghua, Jiangsu, China; 4 Science and Education Department, Xinghua People's Hospital Affiliated to Yangzhou University, Xinghua, Jiangsu, China; 5 Human Resources Department, Xinghua People's Hospital Affiliated to Yangzhou University, Xinghua, Jiangsu, China

**Keywords:** artificial intelligence anxiety, clinical nurses, emotional intelligence, occupational fatigue, structural equation modeling, turnover intention

## Abstract

**Background:**

The rapid integration of artificial intelligence (AI) into healthcare is associated with new psychological challenges. However, the extent of its impact on nursing workforce stability is unclear.

**Aim:**

To explore the relationship between AI anxiety and turnover intention of clinical nurses, and the mediating effect of occupational fatigue and the moderating effect of emotional intelligence (EI).

**Methods:**

A cross-sectional survey was conducted from March to June 2026 among 404 clinical nurses from four hospitals in Jiangsu Province, China. Data were collected using the AI Anxiety Scale (AIAS), Health Workers’ Occupational Fatigue Scale (HWOFS), Turnover Intention Scale (TIS) and Wong and Law Emotional Intelligence Scale (WLEIS). Mediation was tested using structural equation modelling with bootstrap resampling, while moderation was tested using moderated regression analysis.

**Results:**

Differences in baseline turnover intentions across age, department, and professional title level were statistically significant (all *p* < 0.001). Path analysis showed that AI anxiety was positively associated with occupational fatigue (β = 0.611, *p* < 0.001) and turnover intention (β = 0.530, *p* < 0.001). Occupational fatigue partially mediated the association between AI anxiety and turnover intention (indirect effect = 0.299, 95% CI: 0.129, 0.379). EI had a significant negative main effect on occupational fatigue (b = −0.257, *p* < 0.001), but it did not significantly moderate the association between AI anxiety and occupational fatigue.

**Conclusion:**

AI anxiety is significantly correlated with the turnover intention of clinical nurses, and occupational fatigue may represent a potential pathway linking AI anxiety and turnover intention. Although EI is associated with lower levels of occupational fatigue, it fails to cushion the impact of AI anxiety, a specific stressor. Hospital administrators should focus on targeted AI literacy improvement plans and specific fatigue relief strategies to retain clinical nursing staff.

## 1 Introduction

Nurses are at the front line of two pressures that are reshaping healthcare delivery simultaneously: systemic demands to do more with fewer resources (1), and the rapid, largely mandatory adoption of artificial intelligence (AI) across clinical workflows, from medical records (2) and clinical decision-making (3) to infection control (4, 5) and patient services (6). This change does not make the practice of nursing easier, but it is connected to a unique psychological burden. Many nurses report an additional learning burden and fear of technical errors and of being displaced by the very systems designed to help them (7, 8). If nurses see AI as a threat, rather than a support, they are less likely to engage with it; this reluctance may add to their existing workload, rather than relieve it (9, 10). We contend that this AI-related anxiety has emerged as a salient but poorly understood occupational stressor for the contemporary nursing workforce.

The impact of occupational stress in nursing is profound. A survey of 48 public hospitals found that 42.8% of Chinese clinical nurses had turnover intention, and the main reasons were poor work-life balance, sleep deprivation and limited room for career growth (11). The impact is much more than a shortage of workforce in health care facilities: A systematic review of nurse turnover found consistent relationships between adverse patient outcomes such as falls, pressure ulcers and medication errors (12). This proves that turnover is not simply a personal career choice, but a systemic hazard to patient safety. Chronic occupational fatigue is a neglected source of turnover, different from general work-related stress or burnout. Rutledge et al. showed that chronic occupational fatigue was a predictor of turnover intention after controlling for burnout and resilience (13). Early occupational fatigue has also been related to decreased long-term motivation of new nurses (14). These findings suggest a process of gradual resource depletion leading to the decision to leave, identifying occupational fatigue as a more accessible and modifiable intervention point than turnover intention itself.

Despite growing recognition of AI as a workplace stressor, existing nursing–AI research has concentrated almost exclusively on technology acceptance (15), AI literacy (16), and adoption or usage behaviors (17)—that is, on whether and how nurses use AI, rather than on the psychological toll this adoption may exact. What remains unknown is whether AI anxiety, once nurses are already using or anticipating AI in their roles, is associated with workforce-level outcomes such as turnover intention, and, if so, through what potential pathway. Another open question pertains to individual variation in this response. EI buffers general occupational stress (18), but it is unclear whether this protective capacity extends to a stressor as structurally distinct as AI anxiety, which is grounded in threats to professional identity and job security, as opposed to interpersonal demands. This study directly addresses these three gaps by (a) investigating the association between AI anxiety and turnover intention among clinical nurses, (b) testing occupational fatigue as the potential pathway linking the two, and (c) testing whether EI moderates the association between AI anxiety and fatigue.

The expectation that AI anxiety is tied to greater occupational fatigue draws on Conservation of Resources (COR) theory, which holds that people work to build up and safeguard a limited pool of psychological resources, and that losing those resources—or fearing their loss—is itself a source of stress (19). Several features of AI anxiety place it squarely within this kind of chronic stressor. Nurses working alongside AI systems must continually interpret machine-generated outputs, weigh them against their own clinical judgment, and master unfamiliar digital interfaces on top of an already demanding workload, all of which places sustained cognitive load on top of their existing clinical demands (20, 21). Alongside this cognitive burden runs an emotional one: recurring worry about making technical errors, falling behind technologically, or eventually being replaced draws down emotional reserves that would otherwise support day-to-day coping (22). A further, less direct pathway is time itself—adjusting established routines to fit new technology eats into the recovery time nurses would otherwise have. Under COR theory, resource expenditure that outpaces replenishment produces the kind of depletion that surfaces as fatigue, a pattern echoed in prior work showing that other chronic occupational stressors predict fatigue even after burnout is accounted for (13). Taken together, these pathways suggest that AI anxiety may represent a stressor specific to the ongoing digitalization of clinical work, offering both a theoretical and empirical rationale for linking it to occupational fatigue.

Emotional intelligence (EI) is proposed as a moderator of this process, but its role requires distinguishing between two conceptually separate effects. EI has been shown to have a direct, negative association with occupational fatigue: nurses who are more skilled at recognizing and regulating their emotions tend to report lower baseline fatigue, plausibly because effective emotion regulation is thought to lessen the cumulative toll of routine clinical stressors (23). This main effect is distinct from the question of whether EI changes the strength of the specific association between AI anxiety and fatigue—that is, whether EI is expected to buffer nurses against the resource drain associated with AI anxiety. A rationale for this moderating role, beyond EI’s general stress-buffering function, follows from the appraisal component of EI: individuals higher in EI are better able to accurately appraise ambiguous or novel stressors and to deploy adaptive emotion-regulation strategies in response (24). Because AI anxiety arises from a novel, ambiguous, and identity-relevant threat (25), nurses with higher EI may be better equipped to reappraise this threat and regulate the emotional reactions it provokes, which is expected to weaken the association between AI-specific anxiety and resource depletion or fatigue (26). This distinction is important because the two effects suggest different intervention strategies: a main effect would suggest EI training lowers fatigue broadly, whereas a moderating effect would suggest it specifically protects nurses experiencing high AI anxiety.

This study aims to test this integrated model among clinical nurses experiencing AI-driven digital transformation in China. Specifically, the study seeks to examine whether AI anxiety is associated with turnover intention, whether this association is mediated by occupational fatigue, and whether the AI anxiety-fatigue pathway is moderated by EI. This paper’s value lies not only in demonstrating that AI anxiety exists among nurses, but also in tracing the precise route through which AI anxiety is connected to turnover intention via occupational fatigue, and in directly examining whether EI’s general stress-buffering role holds true for this technology-specific strain, rather than assuming that it does. Based on this, we propose the following hypotheses: H1: AI anxiety is positively related to turnover intention in clinical nurses. H2: Occupational fatigue mediates the positive relationship between AI anxiety and turnover intention. H3: EI moderates the association between AI anxiety and occupational fatigue, such that this association is weaker among nurses with higher EI (Figure 1).

**Figure 1:**
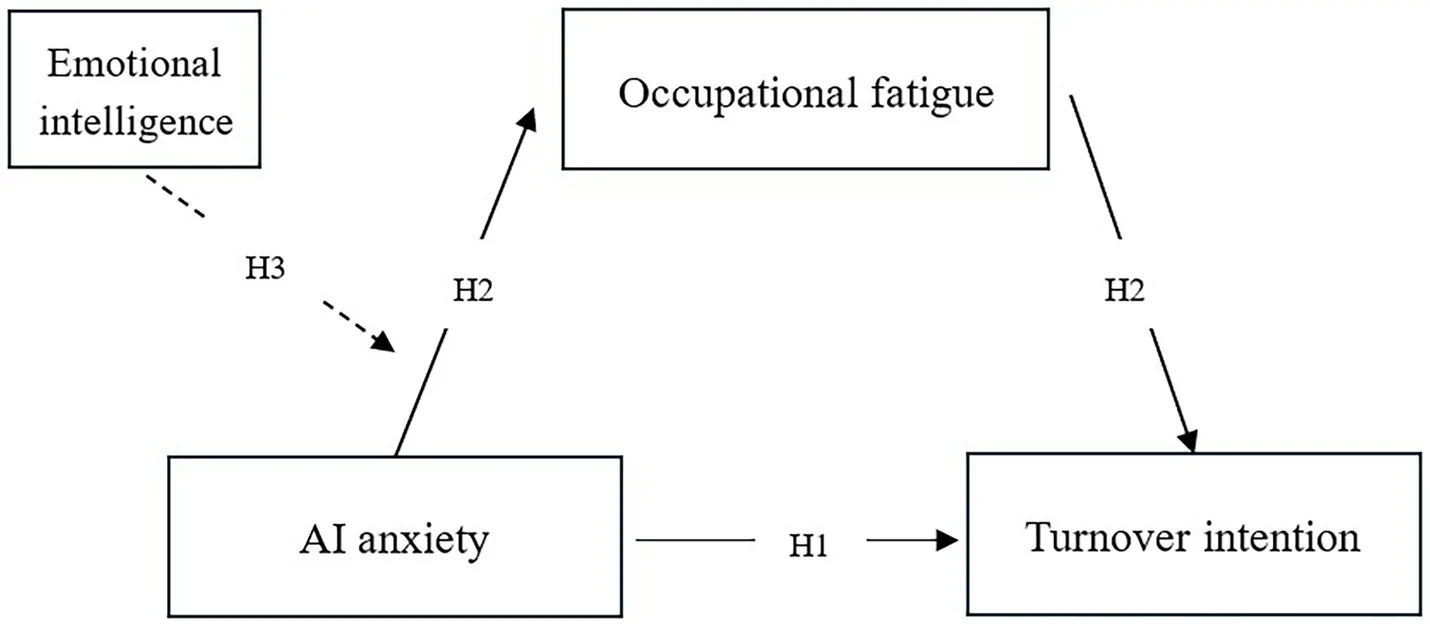
Hypothesized conceptual model. Dashed arrow denotes moderation.

## 2 Participants and methods

### 2.1 Study design and participants

Four public hospitals in northern Jiangsu Province participated in this study, labeled Hospital A (tertiary), Hospital B (secondary), Hospital C (primary), and Hospital D (primary) to preserve institutional anonymity. The cross-sectional study was carried out from March to June 2026. Participants were recruited by convenience sampling. Eligibility criteria for participants were: (1) registered clinical nurses who are working in frontline clinical positions; (2) clinical work experience of no less than 1 year; and (3) informed consent and voluntary participation in the study. Nurses on internship or further training and those who had been absent from work for long periods of time on account of sick leave or maternity leave were excluded. Nurse managers, nurse educators, research nurses, and administrative nurses were also excluded.

According to Kendall’s sample-size guideline, the minimum sample should be 5 to 10 times the total number of variables (27). In this study, the 25 variables comprised 9 demographic and occupational characteristics and 16 dimension-level indicators derived from the four study instruments, yielding a minimum requirement of 260 cases. Because SEM was the primary analytical approach, the required sample size was evaluated considering model complexity, anticipated effect size, statistical power, and significance level (28, 29). Based on an anticipated medium effect size (f^2^ = 0.15), a significance level of 0.05, and a desired statistical power of 0.80, the minimum required sample size was estimated to be sufficient for detecting the hypothesized structural relationships in the proposed SEM model. Considering the four latent constructs, their corresponding observed indicators, and the estimated structural parameters, a target sample size exceeding 300 participants was considered appropriate. To account for 20% invalid responses, the target sample size was increased to at least 360 participants. Ultimately, 404 valid questionnaires were obtained, providing adequate statistical power for SEM estimation and hypothesis testing.

The four participating hospitals are members of a tightly integrated regional medical consortium led by Xinghua People’s Hospital, with the remaining three hospitals comprising one secondary-level and two primary-level institutions. The study protocol was approved by the Ethics Committee of Xinghua People’s Hospital (No. XRYLL-KY-2026009), and this approval covered data collection at all four participating hospitals; Institutional permission for data collection was also obtained from the other three hospitals prior to the survey being distributed.

### 2.2 Measures

#### 2.2.1 General characteristics questionnaire

We collected demographic and occupational characteristics using a self-developed questionnaire. Variables included hospital, age, department, educational level, employment type, professional title, marital status, number of children, and daily working hours.

#### 2.2.2 Turnover Intention Scale (TIS)

Turnover intention was measured using the Turnover Intention Scale (TIS) developed by Michaels and Spector (30). The scale has been widely applied in survey research among clinical nurses in Chinese hospital settings (31). The scale consists of six items covering three dimensions: intention to leave the current job, motivation to seek alternative employment, and perceived possibility of obtaining another job. Items were rated on a four-point Likert scale, with higher scores indicating stronger turnover intention. The scale demonstrated acceptable reliability, with a Cronbach’s α coefficient of 0.818.

#### 2.2.3 Artificial Intelligence Anxiety Scale (AIAS)

AI anxiety was measured using the AIAS scale developed by Wang and Wang (25). The Chinese version of the scale consists of 20 items, covering four dimensions: learning, work substitution, socio-technical blind spots, and AI configuration (32). The higher the score, the higher AI-related anxiety level. The Cronbach’s alpha coefficient of the Chinese version of this scale was 0.941, while in this study, it was 0.964.

#### 2.2.4 Health Workers’ Occupational Fatigue Scale (HWOFS)

Occupational fatigue is evaluated using the HWOFS, developed by Tan et al., which consists of 35 items covering five dimensions: physical fatigue, cognitive-emotional fatigue, social fatigue, sleep-related fatigue, and auditory fatigue (33). This tool is specifically designed for medical workers and incorporates the professional characteristics related to the clinical environment. The higher the score, the more severe the degree of occupational fatigue. In this study, the scale demonstrated good internal consistency (Cronbach’s α = 0.945).

#### 2.2.5 Wong and Law Emotional Intelligence Scale (WLEIS)

EI was measured using the Chinese version of the WLEIS, originally developed by Wong and Law (34). The scale consists of 16 items across four dimensions: self-emotional appraisal, others’ emotional appraisal, emotion regulation, and use of emotion. Responses were rated on a seven-point Likert scale, with higher scores indicating higher EI. The Cronbach’s α coefficient of the scale in the present study was 0.903.

All four instruments were administered using established Chinese versions with documented reliability in Chinese samples; therefore, no additional translation, back-translation, or cultural adaptation procedure was required for the present study.

### 2.3 Data collection and quality control

The questionnaire was distributed electronically via an online survey platform with an estimated completion time of approximately 10 min, and participants could complete the survey on their mobile phone or computer. In each hospital, the survey link was distributed to eligible clinical nurses via the routine internal communication channel of the human resources department and the nursing department; administrators only forwarded the link and did not participate in the identification, monitoring or influencing of participation. Prior to the completion of the questionnaire participants were informed of the purpose of the study, the confidentiality principles and the voluntary nature of the study. Participants were free to decline participation and/or withdraw from the study at any time without consequence. Responses were anonymous and no identifying information was linked to individual submissions and no incentives were given. Each IP address was restricted to a single submission to prevent duplicate entries. The questionnaire was pilot-tested with 10 clinical nurses beforehand, and the fastest completion time observed (180 s) was adopted as the minimum threshold for a valid response; because all items were mandatory, no missing data occurred at the item or questionnaire level.

A total of 446 questionnaires were distributed across the four hospitals. Of these, 42 were excluded based on the pre-specified screening criteria (Figure 2), leaving 404 valid questionnaires and a valid response rate of 90.58% (404/446).

**Figure 2:**
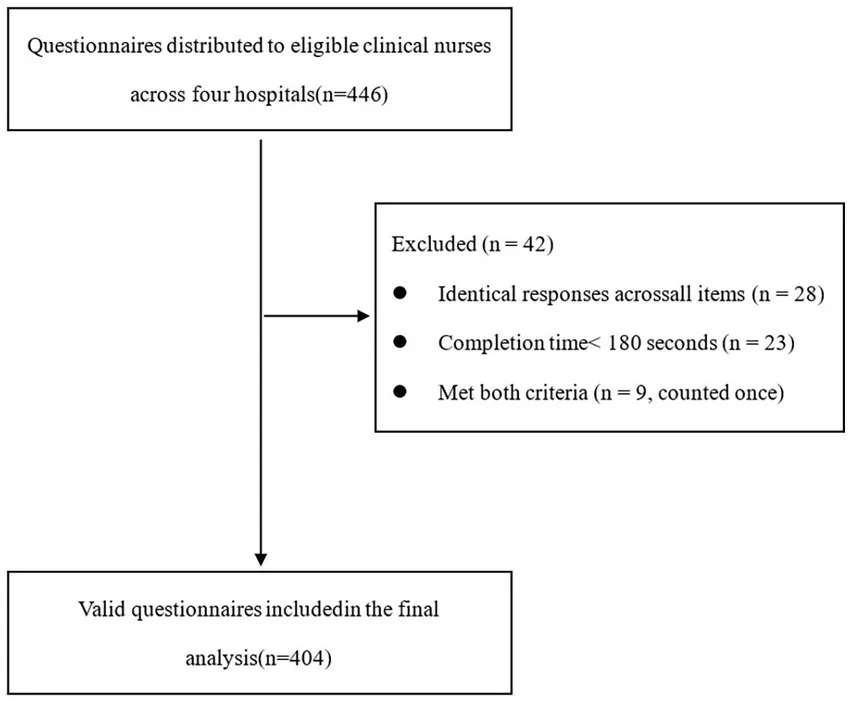
Flow of exclusion and inclusion.

### 2.4 Statistical analysis

Statistical analyzes were carried out using SPSS (version 27.0) and AMOS (version 29.0). Before comparisons between groups, the normality of the continuous variables was mainly assessed with the Shapiro–Wilk test and the Kolmogorov–Smirnov test, together with visual inspection of histograms. The homogeneity of variance across groups was assessed with Levene’s test. Comparison between groups was made using one-way ANOVA when the assumptions of normality and homogeneity of variance were satisfied, otherwise the non-parametric Kruskal-Wallis test was used. For multi-category variables analyzed by Kruskal-Wallis test, post-hoc pairwise comparisons were performed by Dunn’s test to identify the specific groups that were significantly different.

Because all measures were self-reported and collected at a single time point, common method bias was assessed using Harman’s single-factor test and the full collinearity VIF test. Convergent and discriminant validity of the latent constructs were evaluated via confirmatory factor analysis (CFA), examining standardized factor loadings, composite reliability (CR), average variance extracted (AVE).

Pearson correlations and variance inflation factors (VIFs) were used to examine associations and multicollinearity among AI anxiety, occupational fatigue, EI, and turnover intention. Structural equation modeling (SEM) in AMOS was used to estimate the measurement and structural models simultaneously, controlling for age, department, and professional title; model fit was evaluated using χ^2^/df, RMSEA, NFI, TLI, GFI, and CFI. The mediating effect of occupational fatigue was tested via bootstrap resampling (5,000 resamples, bias-corrected 95% CI).

The moderating effect of EI was tested separately using SPSS PROCESS (version 4.2, Model 1), because a latent-variable interaction term could not be accommodated within the SEM framework without more complex estimation methods beyond the scope of this design. AI anxiety and EI were mean-centered before computing the interaction term, with age, department, and professional title entered as covariates; model R^2^ and ΔR^2^ (with corresponding F-tests) were used to evaluate overall and incremental model fit.

For mediation and moderation analyses, significance was determined primarily by whether the bias-corrected 95% CI excluded zero; *p* < 0.05 was used as the significance threshold throughout.

## 3 Results

### 3.1 Sample characteristics and univariate analysis

Of the 404 participants, most worked in tertiary hospitals (63.61%) and clinical wards (82.42%). The majority were aged 25–35 years (49.50%), held a bachelor’s degree or higher (77.97%), and were employed under record-based contracts (71.04%). Baseline turnover intention (TIS) scores differed significantly by age group, department, and professional title (all *p* < 0.001) (Table 1).

**Table 1:** Baseline characteristics and differences in the TIS of participants (*N* = 404).

Variables	Category	*n* (%)	TIS (Mean ± SD)	Statistical value	*p*-value	*Post-hoc* (Dunn’s test)
Hospital	A (Tertiary)	257(63.61)	15.50 ± 2.63	1.602^a^	0.188	-
	B (Secondary)	69(17.08)	16.12 ± 2.55			
	C (Primary)	47(11.63)	15.11 ± 2.82			
	D (Primary)	31(7.68)	15.32 ± 2.66			
Age (years)	< 25	78(19.31)	17.41 ± 1.70	93.651^b^	<0.001	< 25 vs. 25–35^**^; < 25 vs. 36–45^**^; < 25 vs. 46–55^**^; < 25 vs. > 55^**^; 25–35 vs. > 46–55^**^; 25–35 vs. > 55^**^; 36–45 vs. > 55^**^
	25–35	200(49.50)	15.33 ± 2.67			
	36–45	84(20.79)	15.57 ± 2.48			
	46–55	29(7.18)	13.38 ± 1.90			
	> 55	13(3.22)	12.38 ± 1.61			
Department	Clinical	333(82.42)	15.38 ± 2.71	17.947^b^	<0.001	ICU vs. Clinical^*^; ICU vs. Outpatient^*^
	Outpatient	35(8.66)	15.63 ± 2.67			
	Emergency	19(4.70)	16.58 ± 1.07			
	ICU	17(4.21)	17.41 ± 1.62			
Education level	Associate degree	89(22.03)	15.60 ± 2.63	0.195	0.845	-
	Bachelor’s degree or higher	315(77.97)	15.53 ± 2.66			
Employment type	Agency-dispatched	90(22.28)	15.38 ± 2.60	0.247^b^	0.884	-
	Record-based contract	287(71.04)	15.55 ± 2.56			
	Permanent	27(6.68)	16.07 ± 3.58			
Professional title	Junior	174(43.07)	16.28 ± 2.61	15.102^b^	<0.001	Senior vs. Junior^**^ Intermediate vs. Junior^**^
	Intermediate	170(42.08)	15.23 ± 2.56			
	Senior	60(14.85)	14.33 ± 2.39			
Marital status	Married	95(23.51)	15.68 ± 2.09	1.661^b^	0.436	-
	Single	262(64.85)	15.45 ± 2.79			
	Other	47(11.63)	15.81 ± 2.84			
Number of children	0	95(23.51)	15.68 ± 2.09	1.035^b^	0.596	-
	1	256(63.37)	15.51 ± 2.87			
	≥ 2	53(13.12)	15.49 ± 2.44			
Daily working hours (h)	<8	67(16.58)	15.43 ± 3.51	1.530^b^	0.465	-
	8–10	249(61.63)	15.51 ± 2.28			
	>10	88(21.78)	15.75 ± 2.87			

a Analysis of variance (ANOVA) was used when variances were homogeneous.

b Kruskal-Wallis H test was used when variances were heterogeneous.^*^ < 0.05; ^**^ < 0.001.

### 3.2 Measurement model evaluation and common method bias testing

The reliability and convergent validity of the measurement model were evaluated before structural model testing (Table 2). All standardized factor loadings exceeded the recommended threshold of 0.50, indicating satisfactory item reliability. The Cronbach’s α values ranged from 0.818 to 0.966, and composite reliability (CR) values ranged from 0.804 to 0.929, demonstrating good internal consistency. Furthermore, all average variance extracted (AVE) values exceeded 0.50, supporting adequate convergent validity of the latent constructs. These findings indicated that the measurement model had acceptable reliability and validity for subsequent structural equation modeling analyses.

**Table 2:** Standardized factor loadings, average variance extracted (AVE), and composite reliability (CR).

Instrument	Dimension	Cronbach’s α	Loading range	AVE	CR
AIAS	Learning	0.911	0.617–0.885	0.567	0.912
	Work substitution	0.919	0.652–0.903	0.658	0.92
	Socio-technical blind spots	0.863	0.526–0.992	0.638	0.87
	AI configuration	0.884	0.882–0.897	0.792	0.884
HWOFS	Physical fatigue	0.966	0.723–0.891	0.67	0.96
	Cognitive-emotional fatigue	0.928	0.733–0.855	0.651	0.929
	Social fatigue	0.912	0.828–0.882	0.725	0.913
	Sleep-related fatigue	0.888	0.632–0.917	0.672	0.889
	Auditory fatigue	0.845	0.588–0.839	0.596	0.853
WLEIS	Self-emotion appraisal	0.849	0.728–0.804	0.587	0.85
	Others’ emotion appraisal	0.837	0.757–0.900	0.71	0.907
	Emotion regulation	0.891	0.715–0.782	0.562	0.837
	Use of emotion	0.902	0.748–0.873	0.679	0.894
TIS (single-factor)^a^		0.818	0.580–0.812	0.618	0.804

All standardized loadings were significant at *p* < 0.001.

Because all measures were self-reported and collected at a single time point, common method bias was evaluated using several complementary approaches rather than Harman’s single-factor test alone. An unrotated exploratory factor analysis identified a first principal component accounting for 31% of total variance, below the conventional 40% threshold for concern. The correlation matrix among core study variables (Table 3) showed a maximum inter-variable correlation of 0.756, well under the 0.90 level typically taken as evidence of a shared method factor. A full collinearity test based on variance inflation factors was also conducted; all VIFs remained below 1.608, well under the 3.3 threshold. Finally, the four instruments used different response formats (TIS: 4-point; HWOFS: 5-point; AIAS and WLEIS: 7-point). These results suggest that common method bias is unlikely to be a major concern.

**Table 3:** Correlation analysis of the main variables.

Scale	Item count	Score range	Mean ± SD	TIS	AIAS	WLEIS	HWOFS
TIS	6	6–24	15.55 ± 2.65	-			
AIAS	20	20–140	86.98 ± 18.25	0.756^***^	-		
WLEIS	16	16–112	81.93 ± 10.16	−0.668***	−0.518^***^	-	
HWOFS	35	35–175	97.90 ± 25.29	0.693^***^	0.543^***^	−0.490^***^	-

^***^ *P* < 0.001.

### 3.3 Correlation analysis of the main variables

Multicollinearity was minimal, with all VIF values below 1.608 (well within the conventional threshold of 5). Pearson correlations showed that turnover intention was positively associated with both AI anxiety (r = 0.756, *p* < 0.001) and occupational fatigue (r = 0.693, *p* < 0.001), and negatively associated with EI (r = −0.668, *p* < 0.001). AI anxiety was in turn positively correlated with occupational fatigue (r = 0.543, *p* < 0.001) and negatively correlated with EI (r = −0.518, *p* < 0.001) (Table 3).

### 3.4 Structural model fit and mediation analysis

The hypothesized structural model (Figure 3), adjusting for age, department, and professional title, showed acceptable overall fit (Table 4). The incremental fit indices (NFI, TLI, GFI, and CFI) exceeded the 0.8 threshold for acceptable fit but fell short of the 0.9 threshold for excellent fit, and RMSEA, although below 0.10, did not reach the stricter 0.05 benchmark for close fit.

**Figure 3:**
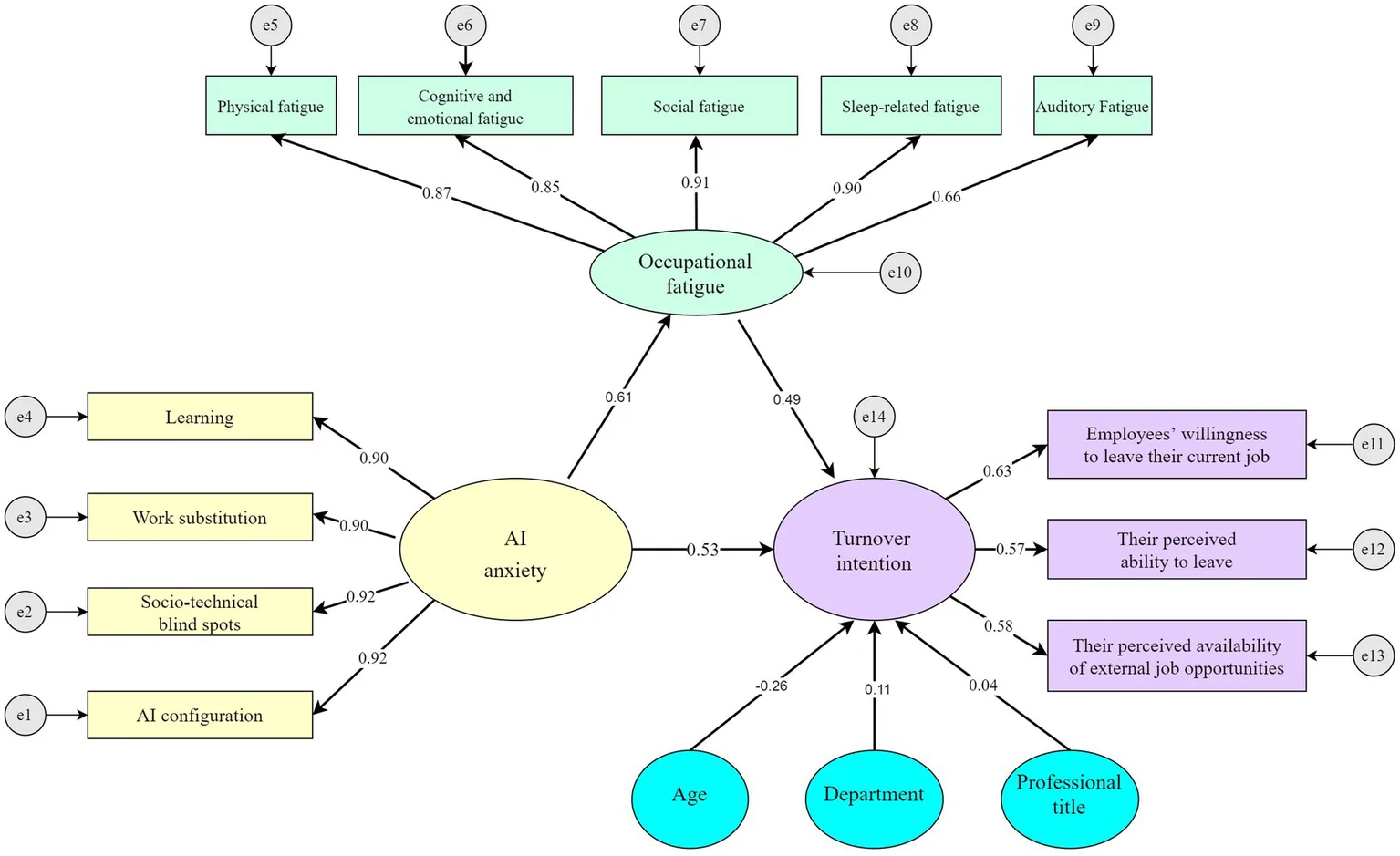
Structural equation model diagram.

**Table 4:** Fit indices of the structural equation model.

**Fit index**	**Recommended criterion**	**Observed value**
χ^2^/df	1–3 = excellent; 3–5 = acceptable	4.323
RMSEA	< 0.05 = excellent; < 0.10 = acceptable	0.082
NFI	> 0.90 = excellent; > 0.80 = acceptable	0.859
TLI	> 0.90 = excellent; > 0.80 = acceptable	0.849
GFI	> 0.90 = excellent; > 0.80 = acceptable	0.846
CFI	> 0.90 = excellent; > 0.80 = acceptable	0.875

All three hypothesized paths were significant (Table 5): AI anxiety was positively associated with occupational fatigue (β = 0.611, 95% CI: 0.539, 0.677) and, both directly and via occupational fatigue, with turnover intention (direct β = 0.530, 95% CI: 0.394, 0.664; occupational fatigue → turnover intention β = 0.489, 95% CI: 0.373, 0.604). These associations remained significant after adjusting for age, department, and professional title. Using bootstrap resampling (5,000 resamples, bias-corrected 95% CI), the indirect effect of AI anxiety on turnover intention through occupational fatigue was significant (β = 0.299, 95% CI: 0.129, 0.379), as was the total effect (β = 0.829, 95% CI: 0.733, 0.934). Because the direct effect remained significant after occupational fatigue was added to the model, these results indicate partial rather than full mediation.

**Table 5:** Results of structural equation modeling and mediation analysis.

Effect type	Path	Standardized estimate (β)	95% CI		*P*-value
			Lower	Upper	
Direct effect	AI anxiety → Occupational fatigue	0.611	0.539	0.677	< 0.001
	Occupational fatigue → Turnover intention	0.489	0.373	0.604	< 0.001
	AI anxiety → Turnover intention	0.530	0.394	0.664	< 0.001
Indirect effect	AI anxiety → Occupational fatigue→ Turnover intention	0.299	0.129	0.379	0.001
Total effect	AI anxiety → Turnover intention	0.829	0.733	0.934	0.001
Covariates	Age → Turnover intention	−0.259	−0.402	−0.126	< 0.001
	Department → Turnover intention	0.105	0.044	0.173	0.013
	Professional title → Turnover intention	0.037	−0.108	0.177	0.376

Structural equation modeling was performed using standardized estimates. Mediation effects were examined using bootstrap resampling with 5,000 iterations, and bias-corrected 95% confidence intervals were calculated. Age, department, and professional title were included as covariates in the structural model.

### 3.5 Moderating effect of EI

AI anxiety and EI were mean-centered before the interaction term was computed. In a moderated regression adjusting for age, department, and professional title, EI was significantly and negatively associated with occupational fatigue (b = −0.257, *p* < 0.001), while the AI anxiety × EI interaction term was not significant (b = 0.043, *p* = 0.187, 95% CI [−0.021, 0.107]) (Table 6). The overall model explained a substantial proportion of variance (R^2^ = 0.602, *F* = 99.921, *p* < 0.001), but the interaction term did not meaningfully improve model fit (ΔR^2^ = 0.0017, *F* = 1.744, *p* = 0.187). H3 was therefore not supported: EI did not significantly moderate the association between AI anxiety and occupational fatigue.

**Table 6:** Moderating effect of EI on the relationship between AI anxiety and occupational fatigue.

Predictor	Coeff	SE	t	*P*-value	LLCI	ULCI
Constant	2.698	0.045	59.54	<0.001	2.608	2.787
AI anxiety	0.219	0.024	9.236	<0.001	0.172	0.265
EI	−0.257	0.027	−9.484	<0.001	−0.31	−0.203
AI anxiety × EI	0.043	0.033	1.321	0.187	−0.021	0.107
Age	−0.105	0.023	−4.619	<0.001	−0.149	−0.06
Department	0.052	0.019	2.711	0.007	0.014	0.09
Professional title	0.043	0.03	1.458	0.146	−0.015	0.102

Outcome variable = Occupational fatigue. Coeff, unstandardized regression coefficient; SE, standard error; LLCI/ULCI, lower/upper limits of the 95% confidence interval.

## 4 Discussion

AI anxiety is more than simply the fact that nurses are using new technology. AI could alter the traditional role of nursing and experience of professional identity for nurses. Nursing is more than a technical skill—it involves clinical judgment, communication, and emotional care. As AI takes on more of the information processing and decision support, some nurses may feel that this broader professional identity is being diminished (35). They also must continue adapting, as AI systems and workflows are always changing. If staff feel unsupported or under-skilled in this process, digital change is felt as a psychological burden rather than an aid at work (36, 37).

Descriptively, the sample’s mean turnover intention score (15.55 ± 2.65, range 6–24) was close to the scale’s theoretical midpoint and nearly identical to the mean reported in a large, same-province survey of Chinese nurses using the same instrument (15.50 ± 3.44) (38), and exceeded the cut-off used elsewhere to classify Chinese nursing samples as showing elevated turnover intention (39). Similarly, the mean AI anxiety score (86.98 ± 18.25, range 20–140) exceeded the scale’s theoretical midpoint and was comparable to levels described as moderate in other recent studies using the same instrument (40). In contrast, the mean occupational fatigue score (97.90 ± 25.29, range 35–175) fell below the scale’s theoretical midpoint.

Our finding that AI anxiety was related to turnover intention suggests that psychological responses to AI are not only related to technology adoption but also to career-related attitudes. Technostress theory offers a relevant explanation: the stress stems from complexity, uncertainty, ongoing learning and changing workflows. AI brings new demands to the clinical setting, such as interpreting AI outputs, adjusting routines, and learning digital resources, which may compound nurses’ anxiety when they doubt their own abilities or perceive limited organizational support. Prior research suggests that AI-augmented environments are associated with increased cognitive load, reduced autonomy, and greater job insecurity (41). Because nursing relies heavily on human interaction, emotional labor, and clinical judgement, a shift toward AI-mediated information processing and advisory functions may be experienced by some nurses as a challenge to professional identity, which in turn may be associated with reduced organizational commitment and a greater inclination to consider leaving (35). Viewed this way, AI anxiety may represent not only an individual adaptation challenge but also a workplace concern during digital transformation.

The central mechanistic finding was that occupational fatigue functioned as a statistical mediator of the association between AI anxiety and turnover intention within this cross-sectional model; because temporal precedence could not be established, this finding should be interpreted as evidence of a plausible potential pathway rather than a confirmed causal pathway. COR theory (42) offers a coherent explanation for this pattern. COR theory holds that individuals seek to protect a finite pool of psychological resources, and that resource loss, or the threat of it, is inherently stressful. Within this framework, the prolonged exposure to demands related to AI (deciphering AI outputs, adjusting established routines, and acquiring new digital devices) is likely to tap into nurses’ cognitive and emotional resources over time, in line with the increased occupational fatigue observed among nurses reporting higher AI anxiety. It is important to note, however, that the present study measured only the endpoints of this proposed process—AI anxiety, occupational fatigue, and turnover intention—and did not directly assess the specific cognitive, emotional, or workload-related resource losses that COR theory proposes as the underlying potential pathway. This account should therefore be read as a theoretically motivated interpretation of the observed statistical mediation, not as a set of empirically measured constructs.

Overall, EI was significantly and negatively associated with occupational fatigue but did not moderate the association between AI anxiety and fatigue. This null result is theoretically informative. The traditional resource model suggests that psychological resources buffer stress, yet our data suggest that AI anxiety—because it involves a perceived threat to job security and professional role—may not be attenuated by individual emotion regulation in the same way that general work-related strain is. EI supports the recognition, expression, and regulation of emotion in interpersonal contexts and has been associated with lower general work-related emotional exhaustion (43, 44), but AI anxiety is structurally different, being driven by perceived skill gaps, shifting role boundaries, reduced job security, and limited organizational backing—concerns that individual emotion regulation alone may not resolve. This result does not imply that EI is unimportant; rather, it suggests that individual psychological resources may help with everyday stress but may not be sufficient to offset strain associated with organizational-level technological change, consistent with recent scholarship advocating a shift from individual adaptation toward “individual–organization co-adaptation” (45).

These findings carry practical implications for the ongoing integration of AI in healthcare. More broadly, AI literacy training may be a useful organizational strategy; such training is likely to be more effective when it addresses not only technical competencies but also AI-related role changes, appropriate reliance on AI outputs, professional autonomy, ethical concerns, and uncertainty regarding job roles, rather than software use alone. Prior literature also suggests that presenting AI as an assisting rather than a replacement technology may help reduce AI-related anxiety (46); because the present study did not directly assess nurses’ perceptions of AI as a replacement threat or their job insecurity, we offer this as a potential intervention strategy for future testing rather than as a finding of this study. Organizational support—including workflow redesign and formal training — is also likely to matter: prior evidence suggests that digital transformation is associated with better outcomes when adequate resources and support are available, and with greater psychological strain when they are lacking (47).

From a public health perspective, our results indicate that psychological strain related to AI, if not addressed, may increase current pressures on nurse retention. Turnover has previously been associated with loss of continuity of care and adverse outcomes for patient safety, including increased falls, medication errors and hospital-acquired infections (48), and the strain related to AI-related stress could, in theory, affect staffing stability, continuity of care, and the overall sustainability of healthcare systems undergoing digital transformation, beyond the well-being of individual nurses. We present this as a plausible implication of our findings, not a directly tested outcome, since staffing and patient-safety outcomes were not measured in this study.

## 5 Limitations

There are some limitations that need to be acknowledged. First, the cross-sectional design does not permit causal inference regarding AI anxiety, fatigue, and turnover intention. Longitudinal studies following changes before and after the introduction of AI would be valuable. Second, our sample was limited to hospitals in one geographic area; sampling across multiple settings would increase generalizability. Third, future research can include other variables, including AI literacy, perceived organizational support, technology acceptance, and professional identity, to develop a more complete model of occupational adaptation in the era of digital health. Fourth, participants were recruited by convenience sampling in hospital nursing departments. This may limit representativeness and introduce selection bias. Although supervisors were not involved in the individual recruitment process and participation was anonymous and voluntary, the fact that recruitment was done through organizational channels may have still influenced the willingness of nurses to participate or the way in which they responded. This possibility cannot be ruled out. Fifth, this study did not include objective indicators of nurses’ actual exposure to AI, frequency of use, technology type, or stage of implementation, so future studies may consider including such indicators to clarify how they relate to AI anxiety and its downstream effects.

## 6 Conclusion

This study suggests that, in the context of digital transformation in healthcare, AI anxiety is an important factor associated with nurses’ turnover intention. Occupational fatigue partially mediated this association in our model, suggesting a possible pathway linking AI-related strain with nurses’ turnover intention. EI was negatively associated with occupational fatigue but did not buffer the specific occupational strain associated with AI anxiety.

## Data Availability

The original contributions presented in the study are included in the article/supplementary material, further inquiries can be directed to the corresponding author.
